# A72 GASTROPARESIS: CLINICAL CHARACTERISTICS, ETIOLOGIES & RESPONSE TO TREATMENT

**DOI:** 10.1093/jcag/gwab049.071

**Published:** 2022-02-21

**Authors:** A Letellier, S Rolland

**Affiliations:** 1 Universite de Montreal, Montreal, QC, Canada; 2 Medicine, University of Ottawa, Ottawa, ON, Canada

## Abstract

**Background:**

Gastroparesis is a gastrointestinal motor disorder characterized by a delay in gastric emptying without evidence of mechanical obstruction. Data pertaining to the demographic and clinical characteristics of gastroparesis in the Canadian population is sparse.

**Aims:**

To review the authors’ experience with gastroparesis.

**Methods:**

This is a retrospective observational study conducted at the *Hôpital Maisonneuve-Rosemont,* a tertiary care center affiliated with the University of Montréal. We collected clinical data from all patients who had a diagnosis of gastroparesis confirmed by gastric emptying scintigraphy from January 2017 to December 2019. The authors reviewed medical records for details on gastroparesis etiologies, response to pharmacologic and non-pharmacologic treatment.

**Results:**

80 patients met the inclusion criteria. 66,8% of them were females. Average age was 62,4 ± 15,7 years. The most frequently identified etiologies of gastroparesis were idiopathic (36.3%), type 1 and 2 diabetes (40%) and medication-induced (23.8%). 82.8% of idiopathic cases were women. 56.6% of patients achieved complete resolution of symptoms with only one pharmacological agent. Response to pharmacologic treatment were mixed, with domperidone and prucalopride having the highest rates of complete resolution of symptoms. Simultaneous use of 2 pharmacologic agents was attempted in 14 patients, half of which achieved resolution of symptoms. All of these patients had at least partial amelioration of their symptoms with the addition of another pharmacologic treatment. Few patients were referred to a dietician, but 66,7% clinically improved with nutritional counselling. Non-pharmacologic approach alone (nutrition consultation, counselling from the physician, withdrawal of causal medication) leads to at least partial amelioration of symptoms in 77,8% of patients.

**Conclusions:**

Diabetic gastroparesis represents the most frequent cause of gastroparesis in our study, with a significant female preponderance. Improvement in symptoms was associated with the use non-pharmacological interventions, as well as simultaneous use of two pharmacological agents.

**Funding Agencies:**

None

